# Assignment of the slowly exchanging substrate water of nature’s water-splitting cofactor

**DOI:** 10.1073/pnas.2319374121

**Published:** 2024-03-04

**Authors:** Casper de Lichtenberg, Leonid Rapatskiy, Michael Reus, Eiri Heyno, Alexander Schnegg, Marc M. Nowaczyk, Wolfgang Lubitz, Johannes Messinger, Nicholas Cox

**Affiliations:** ^a^Department of Chemistry- Ångström Laboratorium, Uppsala University, Uppsala S-75120, Sweden; ^b^Department of Chemistry, Chemical Biological Centre, Umeå University, Umeå S-90187, Sweden; ^c^Max Planck Institute for Chemical Energy Conversion, Mülheim an der Ruhr D-45470, Germany; ^d^Department of Plant Biochemistry, Ruhr-Universität Bochum, Bochum D-44780, Germany; ^e^Research School of Chemistry, Australian National University, Acton ACT 2601, Australia

**Keywords:** photosynthesis, photosystem II, water oxidation mechanism, membrane inlet mass spectrometry (MIMS), electron paramagnetic resonance (EPR)

## Abstract

Identifying the two substrate water sites of nature’s water-splitting cofactor (Mn_4_CaO_5_ cluster) provides important information toward resolving the mechanism of O-O bond formation in Photosystem II (PSII). To this end, we have performed parallel substrate water exchange experiments in the S_1_ state of native Ca-PSII and biosynthetically substituted Sr-PSII employing Time-Resolved Membrane Inlet Mass Spectrometry (TR-MIMS) and a Time-Resolved ^17^O-Electron-electron Double resonance detected NMR (TR-^17^O-EDNMR) approach. TR-MIMS resolves the kinetics for incorporation of the oxygen-isotope label into the substrate sites after addition of H_2_^18^O to the medium, while the magnetic resonance technique allows, in principle, the characterization of all exchangeable oxygen ligands of the Mn_4_CaO_5_ cofactor after mixing with H_2_^17^O. This unique combination shows i) that the central oxygen bridge (O5) of Ca-PSII core complexes isolated from *Thermosynechococcus vestitus* has, within experimental conditions, the same rate of exchange as the slowly exchanging substrate water (W_S_) in the TR-MIMS experiments and ii) that the exchange rates of O5 and W_S_ are both enhanced by Ca^2+^→Sr^2+^ substitution in a similar manner. In the context of previous TR-MIMS results, this shows that only O5 fulfills all criteria for being W_S_. This strongly restricts options for the mechanism of water oxidation.

## Significance

Photosynthesis—the biological process via which solar energy is stored in the form of energy-rich molecules—fuels life on Earth and provides the molecular oxygen we breathe. The crucial starting point for this reaction is the splitting of water, which is carried out by a unique catalyst in Photosystem II. Unraveling the details of this reaction provides the blueprint for how to extract protons and electrons from water using abundant and cheap metal catalysts—a pre-requisite for the sustainable production of green fuels and chemicals. In this study, we identify a key feature of nature’s water-splitting unit, the binding site of one of the two water molecules involved in making O_2_.

**Fig. 1.**
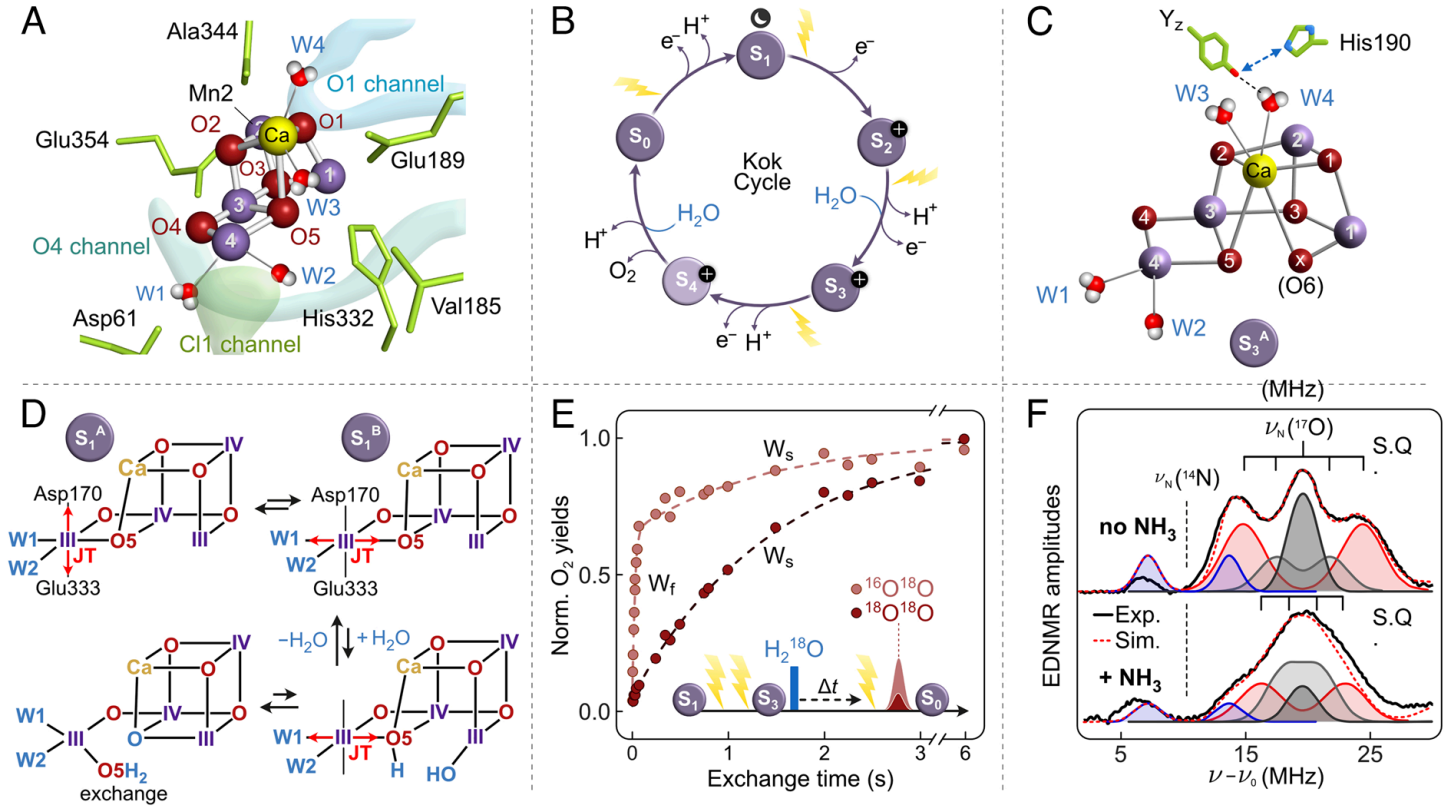
(*A*) The water oxidizing Mn_4_O_5_Ca cofactor with selected ligands and water channels that regulate water access and proton egress. Mn ions are displayed in purple and labeled 1 to 4, oxygen bridges in red and numbered O1 to O5, Ca in yellow, and terminal water/hydroxide ligands are named W1-W4 (based on PDB 6W1O). (*B*) Catalytic (Kok) cycle of the water oxidation reaction (5). S_n_ states (*n* = 0,…,4) indicate oxidation state changes of the Mn_4_CaO_5/6_ cluster. Light-induced electron and proton removal as well as water binding events are indicated. The moon symbol indicates that the S_1_ state is the dark-stable state, and the plus sign an extra positive charge due to lack of proton release from S_1_ to S_2_. (*C*) Schematic view of the Mn_4_CaO_6_ cluster and the tyrosine Z (Y_Z_) – His190 pair in the S_3_ state. The additional oxygen bridge between Ca and Mn1, inserted during the S_2_→S_3_ transition, is known as Ox or O6 (12, 20). Y_Z_ acts as electron relay between the Mn_4_CaO_5/6_ cluster and P680^•+^. (*D*) Proposed equilibria between conformations of the Mn_4_CaO_5_ cluster in its S_1_ state (21). Mn ions are represented by Roman numbers indicating their oxidation state, while oxygen bridges are depicted in red. The red arrow indicates the direction of the Jan–Teller (JT) axis on the Mn4 ion. Below shows one potential pathway for O5 exchange in the S_1_ state via water binding to the Mn1 (22). (*E*) Results of substrate water exchange measurements by membrane-inlet mass spectrometry (MIMS) in the S_3_ state of PSII core complexes from *T. vestitus* at *m*/*z* 34 (^16^O^18^O) and *m*/*z* 36 (^18^O^18^O). The *m*/*z* 34 trace displays a biphasic rise reflecting the rates of the fast (W_f_) and slowly (W_S_) exchanging substrate waters, while the rise of m/z 36 is limited by the exchange of W_S_ (data replotted from ref. 23). (*F*) ^17^O EDNMR spectra (black line) obtained at W-band with highly concentrated PSII samples in absence (*Top*) and after addition (*Bottom*) of ammonia. The blue line signifies the ^14^N coupling arising from the D1-His332 ligand of Mn1, see panel *A* (24). The red dotted line gives the four component fit of the signal (see text). The figure is adapted from ref. 25.

Nature’s water splitting catalyst, a tetra-manganese penta-oxygen calcium cofactor (Mn_4_CaO_5_; Fig. 1*A*) is found in a unique membrane protein complex, Photosystem II (PSII) (1–4). The catalytic cycle of the cofactor is comprised of five distinct redox intermediates, the S*_n_* states, where the subscript indicates the number of stored oxidizing equivalents (*n* = 0 to 4) required to split two water molecules and release molecular oxygen (5) (Fig. 1*B*). Importantly, each S*_n_* state transition is multi-step, with the cofactor’s oxidation coupled to its deprotonation into the bulk and conformational changes (with the exception of the S_1_ to S_2_ transition) (6–15). S*_n_* state progression is driven by the reaction center of PSII, which is a multi-chlorophyll/pheophytin pigment assembly. Light absorption and subsequent charge separation generate an in situ photo-oxidant (P680^•+^), coupled to the Mn_4_CaO_5_ cofactor via an intermediary redox-active tyrosine residue, Y_Z_ (Fig. 1*C*) (1). After four charge separation events, the transiently formed [S_4_] state decays to the S_0_ state with the concomitant release of molecular triplet oxygen and rebinding of one substrate water molecule (14, 15). The term substrate water is used irrespective of the protonation state of the bound oxygen species (16–19).

The dark-stable (S_1_ state) structure of the water splitting cofactor resembles a “distorted chair” with Mn1, Mn2, Mn3, and the Ca^2+^ ion making up the base of the chair, or the open cubane unit as there is no bond between Mn1 and O5 (12, 26, 27) (Fig. 1 *A* and *D*). The outer Mn4 is linked to the open cubane unit via the additional oxygen bridge O4 to Mn3 and by binding to the O5 bridge of the open cubane forming the back of the chair. In the S_1_ state, the oxidation states of the four Mn ions are III, IV, IV, and III (Mn1 to Mn4) (28–30). Recently, two structural isomers were proposed for the S_1_ state (21), S_1_^A^, and S_1_^B^, that are distinguished by the orientation of the Jahn–Teller axis at the outer Mn4(III) ion (Fig. 1*D*). All Mn ions are six coordinate (octahedral) with the important exception of Mn1, which is five-coordinate (square pyramidal), allowing for binding of one water molecule to Mn in the S_2_→S_3_ transition (Fig. 1*B*) as well as O5 exchange (Fig. 1*D*) (12, 18, 20, 22, 31–38). The additional water-derived ligand in the S_3_ state forms a bridge between Mn1 and Ca (Fig. 1*C*) and is known as Ox or O6 (11, 12).

Two substrate waters are required to form O_2_ in the S_3_→S_4_→S_0_ transition. The crystal structures of PSII (26) resolve seven candidates at the Mn_4_CaO_5_ cluster: i) the waters bound to the Ca^2+^ ion (W3, and W4) which form the endpoint of the O1 water channel; ii) the water-derived ligands of the outer Mn4 (W1 and W2) and possibly the oxygen bridge O4 which are connected to the bulk via the Cl1 and the O4 channels; and finally iii) the oxygen bridges O5, and, in the S_3_ state, Ox/O6 (Fig. 1 *A* and *C*) (11, 12, 14, 15, 18, 36, 39–41).

The two substrate waters can be directly monitored using time-resolved membrane inlet mass spectrometry (TR-MIMS). In this experiment, dark-adapted PSII samples are first advanced to the desired S_i_ state using single turn-over flashes. Then, H_2_^18^O is injected and after various incubation times, O_2_ evolution is induced by a sequence of closely spaced flashes. Finally, the isotopic composition of O_2_ is determined by isotope ratio mass spectrometry (16, 18). As the rate of induction of the singly (^16^O^18^O) and doubly (^18^O^18^O) labeled products are significantly different (Fig. 1*E*), the two bound substrates must be ***chemically distinct***. The two substrates are thus referred to as the slowly exchanging substrate (Water slow – W_S_) and the fast-exchanging substrate (Water fast – W_f_) (16). Rates for W_S_ exchange have been measured for all S*_n_* states and vary for *Thermosynechococcus (T.) vestitus* BP-1 (previously *T. elongatus* BP-I) PSII core preparations between 0.4 and 1.1 s^−1^, whereas rates for W_f_ are only well-defined for the higher S*_n_* states (S_2_, S_3_) and are much faster, see Fig. 1*E* (18, 23, 38). In the S_2_ state, the exchange of W_f_ is about 100 s^−1^ and was recently shown to be limited by diffusion of bulk and internal water molecules through the channels leading to the Mn_4_CaO_5_ cofactor (40). Importantly, in the S_3_ state, the exchange of W_f_ is slower (about 40 s^−1^) and the exchange of both substrates is arrested in the S_3_Y_Z_^•^ state (23). Thus, in S_3_ the exchange rate of W_f_ is no longer diffusion limited and hence it is unlikely to be bound to Ca^2+^ as terminal ligand as the rate of ligand exchange for the solvated Ca^2+^ cation is on the order of 10^8^ s^−1^ and thereby much too fast (42). Chemical modification of the cofactor—substitution of the Ca^2+^ ion with Sr^2+^_,_ either by chemical exchange or biosynthetic incorporation—enhances the rate of W_S_ exchange but leaves W_f_ unchanged, suggesting W_S_ is somehow associated with the Ca^2+^ site (23, 38, 43). As terminal Ca ligands are excluded, and since an S*_n_* state dependence of the W_S_ exchange rate is observed, it was proposed that W_S_ could represent an oxygen bridge between the Ca^2+^ ion and a neighboring Mn ion (39, 43). Specifically, the central oxo-bridge was proposed in ref. 39.

For obtaining structural information on exchangeable oxygen ligands a spectroscopic tool is required that is isotope sensitive. Here, in principle, EPR methods are especially suitable owing to their ability to probe the interaction of nuclear spins with the EPR signals of the catalytic site. However, the characterization of oxygen ligands of metallocofactors is challenging owing to the NMR active ^17^O nucleus having a high nuclear spin (*I* = 5/2, quadrupolar nucleus), a small nuclear g-value, and a low natural abundance, with only a handful of published studies using conventional pulse EPR methods (44–48). As a consequence, we developed high field (94 GHz, W-band) ^17^O-Electron-electron Double resonance detected NMR (^17^O-EDNMR), as a sensitive assay for their detection (18, 49). In the high field regime (W-band, 3.4 T), ^17^O ligands of transition metal complexes appear about the characteristic (Larmor) frequency split by the electron-nuclear hyperfine coupling. At this magnetic field, the Larmor frequency of ^17^O [*ν*(^17^O) = 19.6 MHz at 3.4 T] is sufficiently different from background nuclei [e. g. *ν*(^14^N) = 10.5 MHz at 3.4 T] that they can be unambiguously identified (49). The electron-nuclear hyperfine interaction (signal width) can be used to differentiate between different types of ^17^O ligands (O^2−^, OH, OH_2_) (25, 49–51).

Typical EDNMR spectra are shown in Fig. 1*F* (49). Two species are readily observed: i) a background doublet centered at *ν*(^14^N) assigned to the only nitrogen ligand of the cofactor, His332 (Fig. 1*A*) (24), and a broader envelope centered at *ν*(^17^O) assigned to exchanged oxygen sites of the cofactor (49). In our original study, the profile was fitted as three distinct signals: i) a broad signal (splitting ≈10 MHz; red in Fig. 1*F*), which describes the width of the envelope (49). This was assigned to a single, exchanged oxygen bridge (O5), based on comparisons to model systems, and by chemical modification of the cofactor (25, 49–51); ii) a narrow matrix signal (≈1 MHz; dark gray), assigned to exchanged water ligands of the cofactor, dominantly W1; and iii) a third signal of intermediate width (≈4 MHz; light gray) that is hidden under the signal envelope but is better resolved in the double quantum region. This was assigned to a terminal hydroxide ligand of Mn4, W2 (49). Treatment of PSII with ammonia confirms this basic description (lower part of Fig. 1*F*). NH_3_ displaces W1 leading to the suppression of the ^17^O matrix signal, with the central peak broadening as it now has a larger contribution from W2 (25).

Importantly, our initial report demonstrated that all terminal ligands of the cofactor (W1-W4) and O5 exchange within 10 to 15 s with bulk solvent but obtaining exchange rates remained impossible (49). Nevertheless, this structural information from EDNMR further supported our original suggestion made based on the kinetic information from TR-MIMS (39) and point to O5 being W_S_. This was important, since the exchange of an oxygen bridge at such a rate was contested, because in model complexes the rates are much slower owing to the acidity of the μ-oxo bridge motif (51, 52). The reason why O5 likely exchanges rapidly is its conformational flexibility and the neighboring open coordination site at Mn1 that allows extra water to bind and to bring O5, in fully protonated form, to a terminal position for water exchange (Fig. 1*D*) (22, 38).

**Fig. 2.**
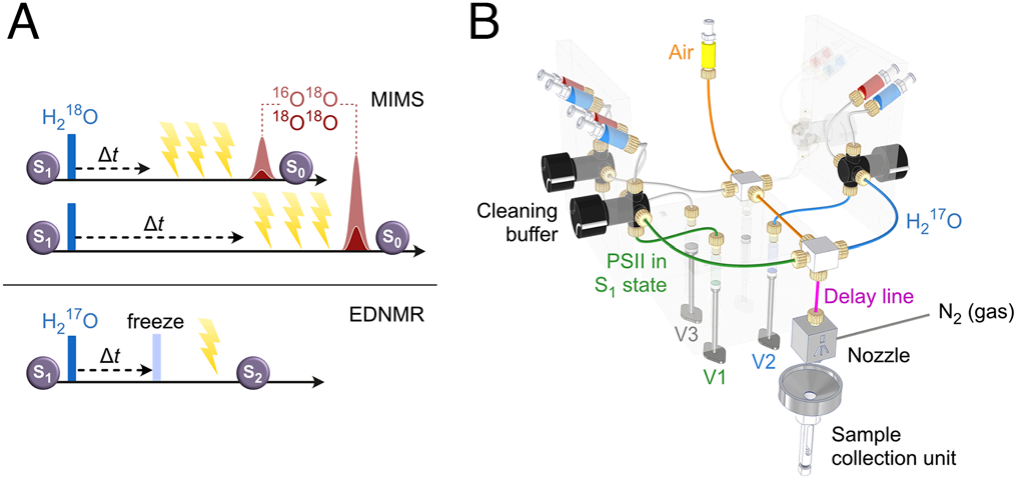
(*A*) Experimental sequences of the water exchange experiments in the S_1_ state of the Mn_4_CaO_5_ cluster employing time-resolved membrane-inlet mass spectrometry (TR-MIMS; *Top*) and time-resolved ELDOR detected NMR (TR-EDNMR; *Bottom*). In both cases, isotope-labeled water (H_2_^18^O resp. H_2_^17^O) is rapidly mixed with the dark-adapted sample. The degree of exchange of the substrate bound at the Mn_4_CaO_5_ cluster with bulk water is then probed after various delay times (Δt). In MIMS, three flashes are given to produce O_2_, which is then analyzed by isotope ratio mass spectrometry, while in EDNMR the exchange process is stopped by freezing and the incorporation of the label is probed by EDNMR after low-temperature (198 K) illumination into the S_2_ state. (*B*) Experimental set-up of the rapid freeze quench system (based on a Biologic QFM 400) for mixing H_2_^17^O (blue) with PSII (green) to study water exchange kinetics at the Mn_4_CaO_5_ cluster in PSII. Specifications: 110 ms deadtime (for PSII), 15 µL sample volume, time resolution determined by delay line (magenta), sample collection on a cold aluminum surface of the collection unit in contact with liquid N_2_, followed by packing into an EPR tube through the central hole of the collection unit.

While EDNMR and MIMS experiments thus far reported strongly point to O5 being the slowly exchanging substrate, they do not conclusively exclude all other water-derived Mn ligands. As such, in the literature, the data have been interpreted to be still consistent with W2 being the W_S_ (53–58) (for discussion see also ref. 38). To resolve this question, we have developed time-resolved EDNMR (TR-EDNMR) that is similar to TR-MIMS (Fig. 2*A*) and allows comparing the exchange rates for O5 and W_S_. Here we demonstrate that these rates are highly similar in Ca-PSII core complexes from *T. vestitus*, and that Ca^2+^/Sr^2+^ substitution significantly enhances the rates of both O5 and W_S_ exchange. Thus, our present data strongly endorse the assignment of the central O5 bridge of the Mn_4_CaO_5_ cluster as the slowly exchanging substrate for photosynthetic dioxygen production.

## Results

### Time-resolved H_2_^17^O Exchange with Freeze-quench.

To determine the rate of O5 exchange we measured the induction of the TR-^17^O-EDNMR envelope as a function of incubation in labeled water employing a purpose-built micro-rapid freeze quench (μRFQ) apparatus (Fig. 2*B*), which is described in detail in *SI Appendix*, Figs. S1–S7 and Text S1), along with calibration data. The system allows the mixing and collection of dark-adapted PSII (S_1_ state) with ^17^O-labeled water, with sample volumes as little as 15 μL, minimizing sample waste—an important consideration when using the expensive ^17^O-labeled water. The ejected sample was frozen on a liquid nitrogen–cooled aluminum surface (funnel), below which EPR capillaries were located. The sample was transferred to the capillary through a hole in the center of the aluminum funnel using a thin rod. It should be noted that the effective concentration of PSII in our μRFQ samples was lower than what we could typically achieve using our standard loading approach, owing to the lower packing efficiency of our capillaries with pre-frozen sample. To combat this, we switched to a higher Q (narrower bandwidth) resonator for all EDNMR measurements, improving overall S/N, but excluding measurements of ^17^O double quantum transitions. Importantly, we can quantify the packing efficiency of each sample tube on the basis of the S_2_ EPR multiline signal induced by 200 K illumination. In this way, we can normalize the entire dataset and thereby compensate for possible sample concentration variations across the set of timepoints.

Detection of the oxygen exchange by EDNMR occurred after a 200 K illumination that quantitatively transferred the sample into the S_2_ state (Fig. 2*A*), which exhibits the S_2_ EPR multiline signal. It is important to note that the signal intensities in the EDNMR experiment are dependent on experimental conditions—and so are not strictly quantitative (59, 60). The length and amplitude of the first microwave pulse of the EDNMR sequence, termed the high turning angle pulse (HTA)—has optimal values for each ^17^O species (18). A short/low amplitude HTA pulse will amplify the species with the largest hyperfine coupling e.g., in this instance O5 and W2, whereas a long/high amplitude pulse will amplify species with smaller hyperfine couplings, e.g., the background ^14^N signal and in particular the matrix signal (W1). That said, as long as experimental conditions are kept constant, then the relative intensity of one signal relative to another is robust allowing kinetic data to be collected (59).

**Fig. 3.**
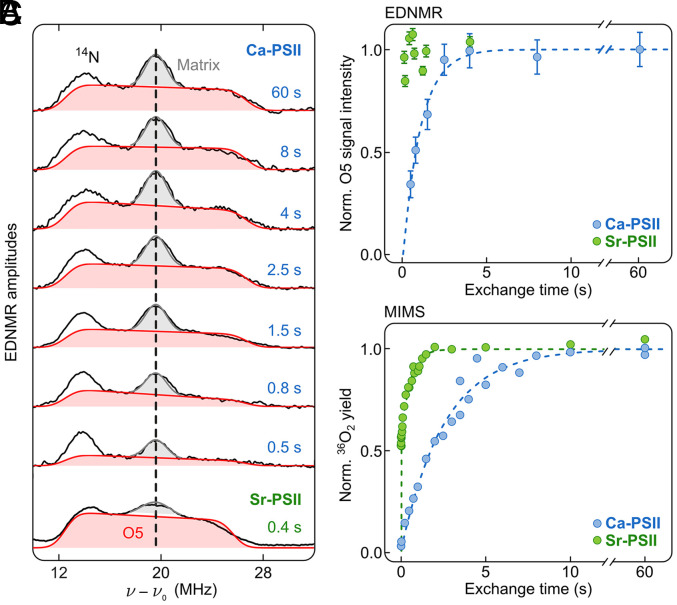
(*A*) Time-resolved ^17^O ELDOR-detected NMR (TR-^17^O-EDNMR) spectra obtained after incubating PSII core complexes from *T. vestitus* at room temperature (22 °C) and pH 6.5 for various times with H_2_^17^O. The first seven traces were obtained with Ca-PSII (60 s to 0.5 s) and the bottom trace with Sr-PSII (0.4 s). Additional spectra are shown in *SI Appendix*, Fig. S9. The integrated intensities of the O5 resonances are indicated (red), and the matrix ^17^O line (gray). Experimental parameters are listed in *Materials and Methods* and the *SI Appendix*, Text S2. (*B*) Exchange time dependence of the integrated O5 resonance in the ^17^O EDNMR spectrum (red area in panel *A*) for Ca-PSII (blue) and Sr-PSII (green). The blue-dashed line is a mono-exponential fit of the Ca-PSII data yielding a rate of 0.9 s^−1^. Error bars were determined by the integrated fitting error, normalized to the overall integral value in the fitting region. (*C*) Normalized ^36^O_2_ flash yields of Ca-PSII (blue) and Sr-PSII (green) core complexes of *T. vestitus* induced by three saturating flashes after different exchange times with H_2_^18^O at 20 °C and pH 6.5. Each dot represents a separate measurement. The dashed lines represent fits of the exchange rates of the slow substrate water (W_S_). A mono-exponential rise with 0.4 s^−1^ was found for Ca-PSII, while a bi-exponential fit with 50% unresolved exchange and a rate of 1.9 s^−1^ for the slower fraction was obtained for the Sr-PSII data.

Fig. 3*A* shows a set of selected TR-^17^O-EDNMR traces collected using different mixing times for PSII core complexes with the natural Ca in the water oxidation complex (Ca-PSII) and of one trace of a sample where Ca was exchanged against Sr (Sr-PSII) (see *SI Appendix*, Fig. S9 for the complete time course of the Sr sample and a replicate dataset for Ca-PSII). The top trace shows the ^17^O envelope seen in Ca-PSII following 60 s incubation in H_2_^17^O. This length of time is longer than required for complete exchange as can be seen from the very similar envelopes obtained at 8 s and 4 s. The spectral profile is essentially the same as reported earlier (Fig. 1*F*). As compared to these earlier studies (49), the ^14^N and the central ^17^O peak are somewhat suppressed relative to the O5 bridge signal due to selecting an initial HTA pulse that maximizes the O5 signal.

To probe the exchange rate of O5, a series of TR-^17^O-EDNMR spectra were collected for Ca-PSII after successively shorter incubation times with H_2_^17^O, down to 0.5 s. The results show a successive decline of the integral O5 EDNMR signal (red) at incubation times shorter than 4 s, such that the O5 EDNMR signal is strongly diminished at 0.5 s (Fig. 3*A*). By contrast, the ^14^N signal, stemming from the D1His332 ligand to Mn1 (24), is observed at approximately the same intensity throughout the time course.

The trace at the bottom of Fig. 3*A* shows the ^17^O envelope seen in Sr-PSII following a 0.4 s incubation with H_2_^17^O. In line with the direct ligation of O5 with Ca/Sr, the O5 signal is slightly narrower (10%) in Sr-PSII as compared to that obtained with Ca-PSII samples, as seen in our previous study (50). Remarkably, the O5 EDNMR signal intensity at 0.4 s is already similar to that in the 60 s trace of Ca-PSII, and to longer incubation times in Sr-PSII (*SI Appendix*, Fig. S9). This shows that the O5 exchange is significantly faster in Sr-PSII than in Ca-PSII.

For both Ca-PSII and Sr-PSII, the central peak, marked in gray, contains information on the exchange rates of W1 and W2 bound to Mn4 (Fig. 1*A*). This central line decreases somewhat at shorter incubation times. However, at this point, we cannot accurately disentangle the exchange of these two water ligands, since the central peak has approximately the same shape across the entire time course (see also Ca-PSII dataset2 in *SI Appendix*, Fig. S9). Reliable analysis is also complicated by the small signal intensity of the central line relative to the O5 signal leading to significant scatter and its variation between datasets. Thus, to characterize these two species (W1 and W2), we need to collect data with much shorter exchange times, and likely switch to ^17^O-ENDOR (51) to reliably decompose the central peak into two components. Both potential improvements are presently challenging to implement (*SI Appendix,* S1).

### Comparison of O5 and WS Exchange Rates.

Fig. 3*B* shows the time course for the induction of the O5 EDNMR signal in Ca-PSII as a function of incubation time with H_2_^17^O water. The intensity of the O5 signal can be estimated from either the maximum of the high field edge of the signal, or by determining the area of the O5 signal by fitting the envelope of the broad rectangular-shaped signal indicated in red in Fig. 3*A*. For better signal-to-noise we employed the latter method, which slightly differs from our original assignment described above, owing to the subsequently found good match of the shape of the broad O5 signal with that seen for exchanged μ-oxo bridges in Mn model complexes and Mn-catalase (49, 51); for further justification see *SI Appendix*, S3 and Fig. S8. Both methods yield a similar induction curve. The resulting data are normalized such that the signal intensity at full exchange is one. The time course of the O5 EDNMR signal rise is well-described by an induction rate of approximately 0.9 s^−1^ (dashed blue line in Fig. 3*B*; see *SI Appendix*, Fig. S10 for a replicate experiment yielding a rate of 1.1 s^−1^). The Sr-PSII data (green symbols) were treated in the same way. They demonstrate that the O5 exchange rate is significantly faster in Sr-PSII than in Ca-PSII – too fast for the determination of an exchange rate with our approach.

For quantitative comparison, the W_S_ exchange rates for Ca-PSII and Sr-PSII were measured by TR-MIMS under nominally the same conditions with regards to pH, buffer composition (*SI Appendix*, Table S4) and temperature employing the same preparations as used for EDNMR (see Fig. 2*A* for the employed flash/injection sequence). The results obtained at the mass-to-charge (*m*/*z*) 36 signal are displayed in Fig. 3*C*. The rise kinetic of this signal reflects the exchange of the more tightly bound substrate W_S_, since for generating ^36^O_2_ both substrates at a catalytic site need to exchange (16, 18) (*m*/*z* 34 data are shown in *SI Appendix*, Fig. S11). The data show that in Ca-PSII the W_S_ exchange rate has a comparable value (0.4 s^−1^) to the O5 exchange (0.9 s^−1^ and 1.1 s^−1^) measured by EDNMR in two independent datasets (Fig. 3*B* and *SI Appendix*, Figs. S9 and S10). We speculate that the 2.5-fold faster O5 exchange seen in TR-^17^O-EDNMR is caused by modest sample heating, from room temperature (22 °C) to about 28 to 30 °C (*SI Appendix*, Text S3.3), owing to friction during the fast mixing of the viscous PSII sample with H_2_^17^O. In contrast, during TR-MIMS experiments, H_2_^18^O is injected with much less friction into a dilute PSII suspension thermostated to 20°C; thus, no temperature increase is expected.

As in the case of O5 exchange in Sr-PSII, the exchange of W_S_ also exhibits a significantly faster rate in Sr-PSII than in Ca-PSII. However, using MIMS, the W_S_ exchange in Sr-PSII can be partially resolved in line with the 2.5-fold slower kinetics compared to TR-^17^O-EDNMR discussed above. Interestingly, the W_S_ exchange in the S_1_ state of Sr-PSII was biphasic, with approximately 50% of centers exchanging at a rate faster than can be measured, while the rate of the second population was approximately fivefold faster (1.9 s^−1^) than the one observed for Ca-PSII. While this biphasic behavior in the ^36^O_2_ data is unusual, we have previously observed it in the *m*/*z* 36 data of substrate exchange experiments in the S_2_-state of Sr-PSII and attributed this observation to a slow conformational equilibrium between a low- and a high-spin form of the S_2_ state (38).

## Discussion

With the aim to structurally identify the slow substrate water W_S_ of the photosynthetic water oxidation reaction, we performed parallel experiments of substrate water exchange employing TR-MIMS and water-ligand exchange at the Mn_4_CaO_5_-cluster using a specially developed TR-^17^O-EDNMR method. These experiments were performed under highly comparable conditions in the S_1_ state on parallel samples of Ca-PSII and Sr-PSII core complexes isolated from *T. vestitus*. This allows a direct kinetic comparison of W_S_ exchange with that of the O5 bridge of the Mn_4_CaO_5_ cluster.

### Assignment of O5 to Ws.

Our present TR-^17^O-EDNMR data (Fig. 3) show that O5 exchanged in the S_1_ state of Ca-PSII with a rate that is very similar to that measured by TR-MIMS for W_S_ exchange. Similarly, the exchange of both O5 and W_S_ was significantly faster in Sr-PSII. Thus, our data unambiguously demonstrate that O5 matches the TR-MIMS characteristics of W_S_. This strongly supports our previous proposal that O5 is the slowly exchanging substrate in all S states (39, 49).

As discussed previously (38), W2 bound to Mn4 is the only other possible assignment for W_S_. The present data do, unfortunately, not allow a direct kinetic analysis of the W2 exchange in the S_1_ state via measuring the double quantum signals, which would allow unobscured observation of W2 exchange (49). This is due to the fact that the experiments had to be performed at lower sample concentrations than our previous experiments in order to allow mixing times with H_2_^17^O that match the W_S_ exchange rates established by TR-MIMS. Thus, our present data alone do not exclude W2 as W_S_; nevertheless, in combination with previous TR-MIMS results this remaining alternative can be eliminated. For this, we discuss in the following two scenarios in which W2 has been proposed to act as W_S_.

In the first scenario, the exchange of W_S_ = W2 would be determined by the isotopic equilibration of both W2 and O5 with bulk water, an arrangement that was proposed to explain the observed S state dependence of W_S_ exchange (54, 61). For the S_1_ state, it was proposed that O5 exchanges via W2 and thereby markedly slows the observable exchange rate of W2 [therefore the authors formally designated O5 as W_S_ (54, 61)], while in S_2_ and S_3_ only W2 can exchange. First, we contest the kinetic viability of this model, since a slow exchange of O5 with W2 cannot markedly slow the much faster exchange of W2 with bulk water. Second, this proposal does not straight forwardly account for the Ca/Sr dependence of W_S_ exchange, since W2 has no direct connection with Ca. Finally, in this (54, 61) and other nucleophilic attack proposals (54, 57, 58, 61) W_f_ is assigned to W3 that remains bound to Ca in the S_3_ and S_4_ states. Thus, these proposals struggle to account for the slowing of W_f_ exchange during the S_2_→S_3_ transition, and cannot explain the arrest of W_f_ exchange in the S_3_Y_Z_^•^ state (18, 23).

In a second scenario, W_S_ is assumed to be W2 in the S_0_ to S_2_ states where its exchange rates would be modulated via S state–dependent conformational equilibria (38, 62). In contrast to scenario 1, W2 may then rotate during the S_2_→S_3_ transition via a pivot/carousel water insertion at Mn4 into the original O5 binding site (63, 64). In such a scenario, O-O bond formation may occur between the original W2, now the central oxo-bridge, and the original O5, which is proposed to be pushed by this water insertion path into the Ox/O6 binding site (Fig. 1*C*) (63, 64). Therefore, in the S_1_ state, W_f_ would be O5, which is in conflict with the present data that yield, in the S_1_ state, an exchange rate for O5 that is very similar to that measured by TR-MIMS for W_S_ and thereby more than 100-fold slower than that of W_f_ (*SI Appendix*, Fig. S11 and refs. 38 and 40).

In conclusion, the combination of TR-^17^O-EDNMR and TR-MIMS demonstrates that O5 is the only viable candidate for W_S_.

### Biphasic W_S_ exchange in the S_1_ state of Sr-PSII.

The exchange of W_S_ in Sr-PSII measured by TR-MIMS displayed a biphasic behavior, with an equal distribution between an unresolved fast phase and a resolved slower phase. Compared to Ca-PSII, the W_S_ exchange in the resolved part of Sr-PSII was nearly fivefold faster.

The biphasic exchange of W_S_ in the S_1_ state of Sr-PSII may indicate the presence of two S_1_ state structures. Evidence for two S_1_ structures was reviewed by Pantazis (4). More recently, Drosou et al. (21) proposed that the two S_1_ state conformations differ in the orientation of the JT axis at the Mn4(III) ion: in the more stable S_1_^A^ state the JT axis is proposed to be along the coordination axis of the D1-Asp170 and D1-Glu333 ligands, while in the less stable S_1_^B^ the JT axis is oriented along the W1-Mn4-O5 axis (Fig. 1*D*). Although this proposal awaits experimental confirmation, we propose that these two S_1_-state conformations provide an explanation for the two W_S_ exchange rates. Two cases are possible, both assume that the two conformers are close in energy (thus populated about 50% each) and that S_1_^B^ state facilitates fast O5 exchange. In case 1, the two rates reflect the O5 exchange rate in S_1_^A^ and S_1_^B^, respectively. This assumes that there is a high barrier for the conversion of conformer S_1_^A^ to S_1_^B^. In case 2, which we consider more likely, the faster rate reflects the exchange in the S_1_^B^ state, while the slower rate reflects the conversion of S_1_^A^ to S_1_^B^. In this scenario, the faster W_S_ exchange in the S_1_^B^ state can be understood on the basis of a proposal made for O5 exchange in the S_1_ state (22), in which an extra water molecule needs to bind to the open coordination site of Mn1 (Fig. 1*D*). This rate-limiting step involves a proton transfer to O5 that will be energetically more favorable in the S_1_^B^ state where the JT axis is oriented toward O5 as this makes O5 a better proton acceptor. Consequently, the new water molecule can replace the O5 bridge more easily, which makes O5H to a terminal ligand of Mn4—a prerequisite for further protonation and its exchange with bulk water.

The slower, monophasic O5 exchange in Ca-PSII as compared to Sr-PSII may then indicate that both the energy difference between the A and B forms of the S_1_ state as well as the barrier for conversion of S_1_^A^ to S_1_^B^ are slightly larger in Ca-PSII. This would lead to nearly 100% S_1_^A^ population and the observed 5-fold slower conversion into the still rapidly exchanging S_1_^B^ conformation.

### Consequences for the Mechanism of O-O Bond Formation.

**Fig. 4.**
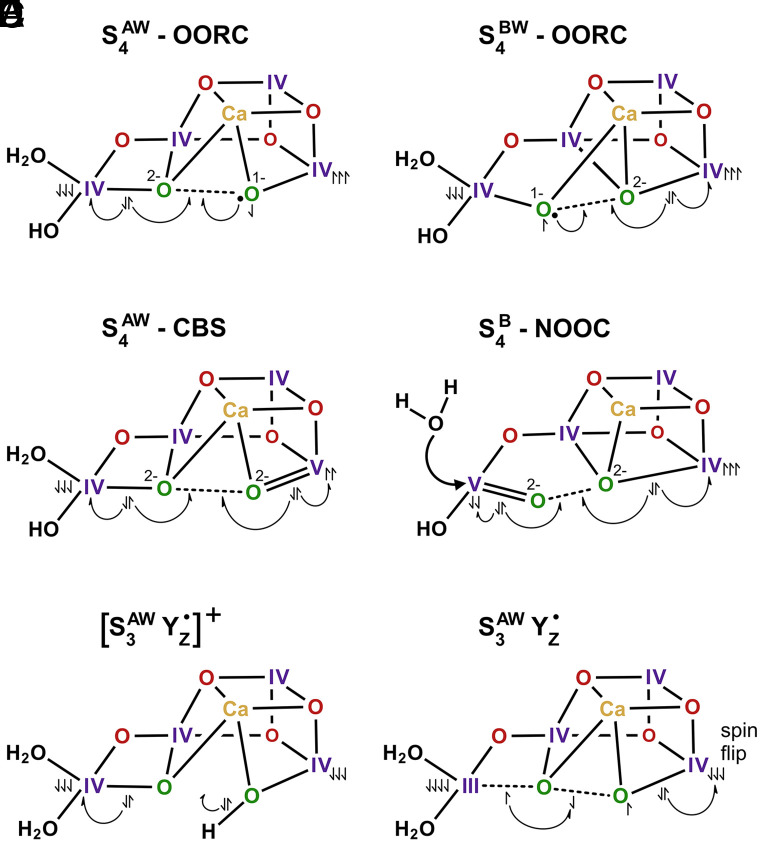
Comparison of selected mechanisms for O-O bond formation in photosystem II that includes O5 as a substrate. In all cases (*A*–*F*), a sketch of the calculated structure of the transient S_4_ or S_3_Y_Z_^•^ state is shown from which O-O bond formation is initiated. S_4_^AW^ states (*A*, *C*, *E*, and *F*) are open cubane (or “right open”) structures that have taken up one substrate water into the open binding site at Mn1 during the S_2_→S_3_ transition. The position of O5 and the identity of the other substrate oxygen relative to the S_1_ state structure (Fig. 1*A*) depend on the water insertion mechanism, which remains controversial (for review, see refs. 4 and 74). Thus, no distinction is made between the slow- and fast-exchanging substrate water. For clarity, both substrate oxygens are shown in green. The two closed cubane (or “left open”) structures of the S_4_ state (*B* and *D*) are denoted as S_4_^BW^ and S_4_^B^, respectively. While S_4_^BW^ has taken up one substrate water during the S_2_→S_3_ transition, for S_4_^B^ it is assumed that water binding does not have to occur up to the S_4_ state (75). The two oxo-oxyl radical coupling mechanisms (*A* and *B*) are denoted as OORC, the concerted bond switching mechanism (*C*) as CBS, and the nucleophilic oxo-oxo coupling (*D*) is signified as NOOC. Structures (*E*) and (*F*) are intermediates of a not named O-O bond formation mechanism that starts from a higher spin state (*S* = 6) and is proposed to occur prior to oxidation of the cluster by Y_Z_^•^, which is achieved by sequential electron donation to two Mn(IV) ions. Note that there is a proton release between *E* and *F* and that peroxide formation starting from *F* requires a flip of the unpaired spins on Mn1 (69, 70). For better comparability and for emphasizing the similarity of the five proposals, the unpaired spins at Mn4 are denoted in all cases as spin down, while those on Mn1 as spin up (except *E* and *F*). Similarly, the bent half-arrows indicating the electron movements upon peroxide formation have been adjusted from the original proposals. Substrate oxidation in the OORC mechanisms (*A* and *B*) is indicated by a black dot near a green O. *A*, S_4_^AW^–OORC (65, 66); *B*, S_4_^BW^–OORC (ref. 66; based on ref. 39); *C*, S_4_^AW^–CBS (67, 76); *D*, S_4_^B^–NOOC (ref. 68; based on ref. 75); (*E* and *F*): [S_3_^AW^ Y_Z_^•^]^+^ and S_3_^AW^ Y_Z_^•^ (69, 70); based on refs. 71–73.

Presently, a number of different mechanisms are discussed for O-O bond formation, which involve W2, W3, O5 and/or Ox/O6 as substrates. Our study strongly favors those mechanisms that assign O5 as the slowly exchanging substrates. In contrast, our present data provides no support, for example, for O_2_ formation via nucleophilic attack of W2 by W3 (54, 55, 57, 58). Nevertheless, a number of mechanisms remain, since the fast-exchanging substrate water has not been uniquely identified. At present, the water-derived ligands at the W2 and Ox/O6 binding sites of the S_3_ state both remain acceptable assignments on the basis of substrate water exchange results that indicate that the fast substrate is bound to Mn in the S_3_ state (discussed in ref. 40). Fig. 4 depicts the prospective S_4_ or S_3_Y_Z_^•^ states of five such proposals, drawn from current computational studies (65–71). It is remarkable that despite differences in geometry of the cluster or lack of water binding during the S_2_-S_3_ transition, in all cases a near linear Mn4-O O-Mn1 arrangement is found. Proposals A-D have in common that the spins on Mn4 and Mn1 are antiparallel, owing to the coupling within the Mn_4_CaO_6_ cofactor, which allows triplet ^3^O_2_ formation without the need for a spin conversion (65). In model E a higher spin state (*S* = 6) is utilized in which Mn4 and Mn1 have parallel spins to form a [O-O]^3−^ intermediate (model F) at the level of the S_3_Y_Z_^•^ state by reducing Mn4. Note that formation of the peroxide intermediate, [O-O]^2−^, from F requires flipping the unpaired spins on Mn1 to allow its reduction. Thus, also in this case an antiparallel spin arrangement between Mn1 and Mn4 is required to form a peroxide intermediate (72, 73). We suggest that the options displayed in Fig. 4 capture the key design features needed for efficient Mn-based water oxidation, both in biology and by synthetic catalysts.

## Summary

In this study, we developed the H_2_^17^O/H_2_^16^O exchange TR-EDNMR approach and used it to show that O5 is the slowly exchanging substrate of water oxidation in PSII based on direct kinetic comparison to W_S_ exchange kinetics measured by TR-MIMS under highly similar conditions. Our finding limits eligible proposals for the dioxygen formation/release mechanism to a set of five highly related options, all based on a similar O-O bond formation geometry and spin coupling of the manganese cluster. By adding flash illumination and active temperature control, the new TR-^17^O-EDNMR technique is expected to allow studies of H_2_^17^O exchange also in the S_2_, S_3_, and S_3_Y_Z_^ox^ states that may eventually lead, together with snap-shot crystallography at XFELs (14) and TR-FTIR (15), to a further refinement of the experimental identification of the mechanism of this important enzymatic process.

## Materials and Methods

PSII core complex preparations from *T. vestitus* were obtained as described earlier (74, 77, 78) and stored at −80 °C at a chlorophyll concentration of 2 to 3 mg/mL (pH 6.5; for details, see *SI Appendix*, Text S2 and section 2.1).

### TR-MIMS ^18^O/^16^O Exchange Measurements.

PSII samples were thawed on ice, diluted to 0.3 mg/mL Chl and poised at S_1_ by a pre-flash and dark incubated at room temperature for 1 h. The substrate water exchange measurements in the S_1_-state of the Kok-cycle were performed at 20 °C essentially as described previously (16, 38). For minimizing injection artifacts, dissolved oxygen in the ^18^O labeled water (97% ^18^O) was removed by addition of a mix of glucose, glucose oxidase, and catalase prior to rapid mixing of the labeled water with Ca- or Sr-PSII. Flash and injection timing was controlled with trigger pulses generated by a LabView routine, while O_2_ and Ar isotopologues were detected with an isotope ratio mass spectrometer (Finnigan Delta plus XP). See *SI Appendix*, Texts S2 and S3 for further details.

### TR-EDNMR Measurements.

PSII samples were thawed, and then concentrated to 4 to 5 mg Chl/mL (EDNMR set 1) or 8 to 9 mg/mL (EDNMR set 2) and loaded into the freeze quench system. Samples were mixed with ^17^O water (set 1: 77% enriched containing buffer with glycerol; set 2: 86%, no additions); see *SI Appendix*, Table S4 for the buffer composition during the exchange experiments. After freeze quenching, the frozen samples were packed into the EDNMR tubes. The S_2_ state was generated by illuminating the PSII samples for 3 s at 198 K (ethanol–dry ice bath) with two 250 W halogen lamps filtered by a 2 cm CuSO_4_ solution (5% w/v) and two filters each: Schott KG 3 (2 mm) and Schott GG 445 (2 mm). The final light intensity at the sample level was about 0.5 W/cm^2^.

EDNMR measurements at W-band were performed on these samples using a Bruker ELEXSYS E680 spectrometer at *T* =4 .8 K. Electron spin echo detected field-swept EPR spectra were measured using the pulse sequence: t_p_ -τ-2 t_p_ -τ- *echo*, with t_p_ = 20 ns and *τ* = 600 ns. EDNMR spectra were collected using the pulse sequence: t_HTA_ -T- t_p_ -τ- t_p_ -τ- *echo,* with t_HTA_ = 6 μs, t_p_ = 80 ns, *τ* = 600 ns, and T = 1 μs. Simulations of the EPR and EDNMR spectra were performed using the EasySpin package (79). See *SI Appendix*, Texts S2 and S3 for further details.

## Supplementary Material

Appendix 01 (PDF)

## Data Availability

All study data are included in the article and/or *SI Appendix*.
