# Supplementary material for: Assignment of the slowly exchanging substrate water of nature’s water-splitting cofactor
Source: Proc Natl Acad Sci U S A. 2024 Mar 4;121(11):e2319374121. doi: 10.1073/pnas.2319374121 (PMC10945779; doi:10.1073/pnas.2319374121)
Supplement: Supplementary file 1 — Appendix 01 (PDF) [file pnas.2319374121.sapp.pdf]

Classification: Chemistry (major); Biophysics and Computational Biology (minor)

Title: **Assignment of the slowly exchanging substrate water of Nature's water-splitting cofactor**

Running Title: Slowly exchanging substrate of Nature's water-splitting cofactor


Authors:

[†,a,1]Casper de Lichtenberg, [‖,1]Leonid Rapatskiy, [‖]Michael Reus, [‖,b]Eiri Heyno, [‖]Alexander Schnegg, [‡,c]Marc M. Nowaczyk, [‖,]*Wolfgang Lubitz, [†,a,] *Johannes Messinger and [‖,¶,]*Nicholas Cox

Author affiliations:

[†]Department of Chemistry – Ångström Laboratorium, Uppsala University, 75120 Uppsala, Sweden; [a]Department of Chemistry, Chemical Biological Centre (KBC), Umeå University, S-90187 Umeå, Sweden; [‖]Max Planck Institute for Chemical Energy Conversion, Stiftstr. 34-36, D-45470 Mülheim an der Ruhr, Germany; [b]current address: Research School of Chemistry, Australian National University, Acton ACT, Australia; [‡]Ruhr-Universität Bochum, Universitätsstr. 150, D-44780 Bochum, Germany; [c]current address: Institut für Biowissenschaften, Abt. Biochemie, Universität Rostock, Albert-Einstein-Straße 3, D-18059 Rostock, Germany; [¶]Research School of Chemistry, Australian National University, Acton ACT, Australia

[1]These authors contributed equally to this work

*To whom correspondence should be addressed: wolfgang.lubitz@cec.mpg.de, johannes.messinger@kemi.uu.se, nick.cox@anu.edu.au

Corresponding authors:
Wolfgang Lubitz
Phone: +49 208 306 3508
E-Mail: wolfgang.lubitz@cec.mpg.de

Johannes Messinger
Phone: +46 18 471 3671
Email: johannes.messinger@kemi.uu.se

Nicholas Cox
Phone: +61 2 6125 8128
E-mail: nick.cox@anu.edu.au



Keywords:, Photosynthesis, Photosystem II, water oxidation mechanism, substrate water exchange, Electron Paramagnetic Resonance (EPR), Membrane Inlet Mass Spectrometry (MIMS)

## SI TEXT

### S1. Purpose-built micro-volume rapid freeze quench (μRFQ) apparatus

**S1.1 *Modifications to BioLogic QFM400 instrument***. The bespoke micro-rapid freeze quench (μRFQ) apparatus developed in this study is based on a BioLogic QFM400 instrument. This was originally designed to perform quench-flow experiments. The system has four built-in working volumes, with independently controlled plungers via stepping motors. Solutions are loaded into the instrument using four position valves. These are used to select the traditional quench-flow mode, or the extra-small volume mode this instrument is capable of. The mixers are designed to provide turbulent mixing over a very large range, so that a single delay line can be used for all ageing times from 4 ms up to several seconds.

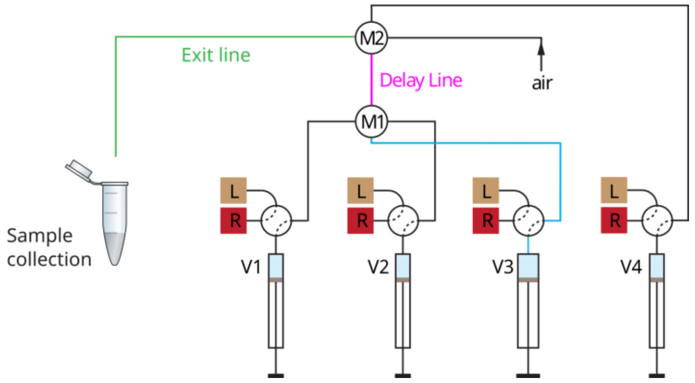


**Fig. S1.** Inlet/outlet ports of BioLogic QFM400 operating in the standard quench flow configuration.

In the typical quench-flow experiment (see **Fig. S1**) the reaction starts after mixing reagents from the working volumes V1 and V2. The reaction evolves in the delay line and stops following injection of the quenching reagent from the volume V4. The reaction only occurs between mixers M1 and M2. The aged solution is then flushed through an exit line into a collect vessel. The volume V3 is usually filled with buffer for washing the delay line between experiments. Pressurized gas can be also used to remove remaining liquids from the exit line. All plungers are driven by stepping motors individually and the speed is calculated depending on the selected sequence in the Biokine software. The maximum flow rate is limited to 1 mL/s for each syringe.

To operate as a micro-volume freeze quench apparatus, the BioLogic QFM400 required modification of the mixing scheme, which is shown in **Fig. S2**. This configuration was adopted to decrease the total volume required per sample, per experiment. This is achieved by mixing the reactants V1 and V2 in a low volume (30μl) delay line. The mixture is then incubated during selected delay times, and is immediately sprayed through the nozzle onto a cold metal surface in contact with liquid $N_2$. A pressured $N_2$ gas line is connected to the nozzle, and is used for spraying the sample even at low jet speed, equalizing the flight time and average particle size. Thus, the freezing time can be estimated as an equal offset independent of the delay time on the kinetics curve. The external electric valve that opens/shuts the $N_2$ gas is controlled by the Biokine software.

The synchronization of the spraying unit with the mixing scheme is made possible by externally triggering the BioLogic QFM400 unit. This is achieved using the SYNC OUT port in the MPS-70 controller unit, which starts the preprogrammed sequence. When the spraying unit is triggered from the sequence, it fires a 24 $V_{DC}$ 2-way solenoid valve SV (Sensor Technics Series 9, serial number 009-0172-900, up to 86 bar).

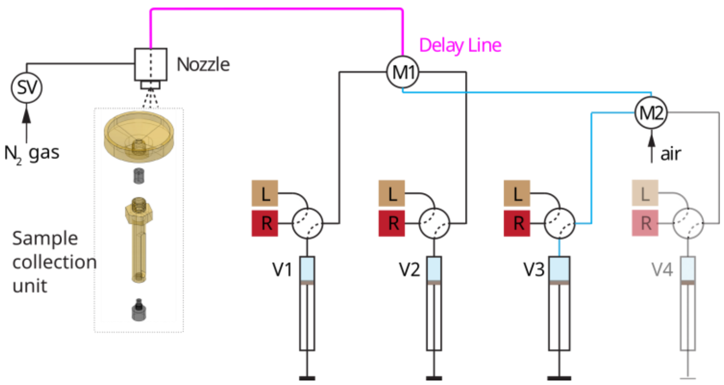


**Fig. S2.** Inlet/outlet ports of BioLogic QFM400 operating in the modified micro-volume freeze quench configuration. The volumes V1 and V2 are filled with the desired reactants (here: PSII and $H_2{}^{17}O$ or TEMPOL and sodium dithionite for calibration), while V3 is filled with the cleaning solution.

The dead volume of the mixing device corresponds to the volume of tubing and junctions from the loading port to the mixer M1, filling the junction to the working volume V1 as well. The detailed scheme of tubing is shown in **Fig. S3**. The calculated tubing volume is $V_T = 39$ μL. Including the volumes of the valve and the mixer M1, the dead volume can be estimated as $V_D \leq 50$ μL.

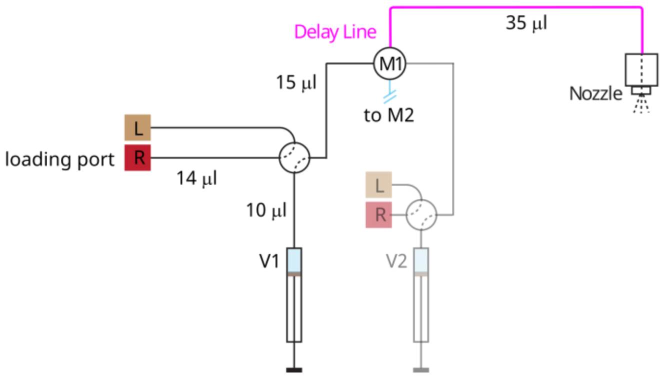


**Fig. S3.** Detailed mixing scheme with calculated tubing volumes for each element.

The mixed solution exits the nozzle through a Hamilton needle gauge 22s (I.D. = 0.168 mm). This was cut to 10 mm length and the point was flattened, then it was inserted in the spraying unit.

The specifications for all 1/16 inch HPLC tubing in the freeze-quench configuration was as follows: the air purging line tube made of PEEK tubing (I.D. = 0.762 mm), the delay line made of ETFE (I.D. = 0.762 mm), and other tubing made of ETFE (I.D. = 0.508 mm).

The configuration shown in Fig. S3 defines the minimum mixing time, which is limited by the highest possible flow rate. For the given 35 μL delay line it is 18 ms.

The sample collecting system for W-band quartz tubes is made of aluminum and similar to one that was used by Schünemann et al. (1). A similar collecting system was also designed for X- and Q-band EPR tubes. The packing process leads to sample losses that were estimated for different EPR tube sizes based on average collected sample volumes (see **Table S1).**

**Table S1**. Dimensions of EPR tubes and corresponding sprayed and collected volumes per time point.

| | *X-band* | *Q-band* | *W-band* |
|---|---|---|---|
| **EPR tube i.d.** | 2.8 mm | 1.8 mm | 0.6 mm |
| **EPR tube o.d.** | 3.8 mm | 2.8 mm | 0.84 mm |
| ***V* sprayed per sample** | 2x75 µL | 2x30 µL | 2x15 µL |
| ***V* collected in the tube** | 120 µL | 40 µL | 4 µL |

The entire unit is shown in **Fig. S4**.

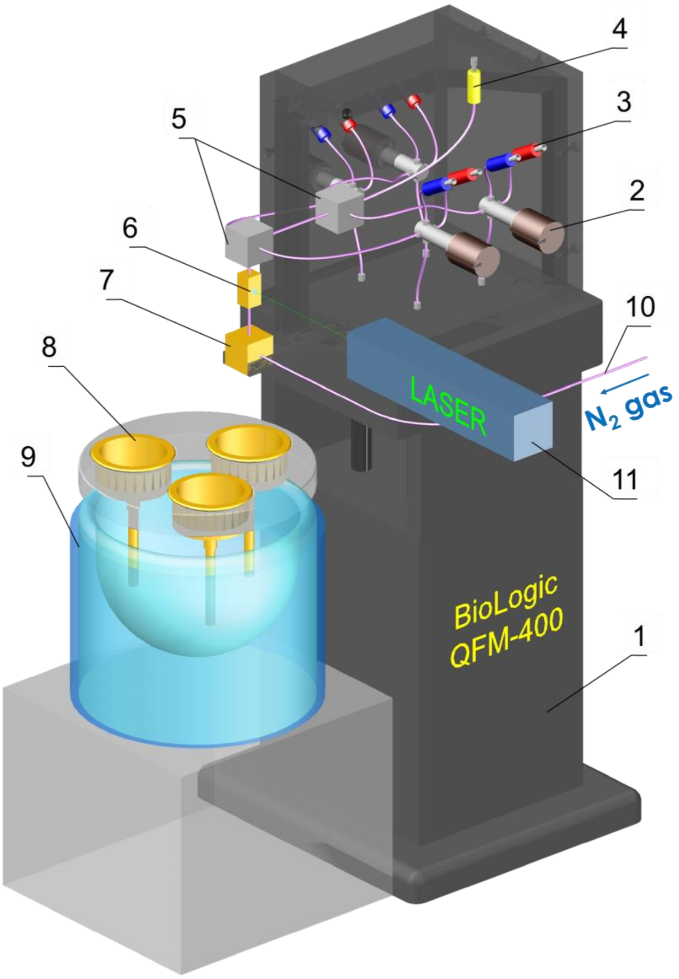


**Fig. S4**. Drawing of the BioLogic QFM-400 instrument, modified for RFQ application: 1) mixing module; 2) 4-way valve; 3) loading port; 4) air-port; 5) mixing blocks; 6) optic port; 7) nozzle spraying block; 8) sample collection system; 9) liquid $N_2$ dewar; 10) $N_2$ gas line; 11) pulse laser (not used in this work).

To test the performance of the µRFQ apparatus on EPR measurable samples, the reaction of the stable radical TEMPOL (4-hydroxy-2,2,6,6-tetramethylpiperidin-1-oxyl) with the two reductants: sodium dithionite (2) and sodium ascorbate in excess were employed. These two well described reactions cover different timescales, from the millisecond range through to the second range.

**S1.2 *Calibration and performance testing of µRFQ apparatus – short timescale regime (<100 ms).***

The time-course of the reaction of TEMPOL with sodium dithionite is shown in **Fig. S5.** These experiments were performed using a 10% v/v glycerol/water solution to match the viscosity of the target protein samples. The experiments were performed using W-band capillaries of the same dimensions as for the target proteins. The TEMPOL radical is observed at approximately 3.352 T and, at W-band, resembles a broad Gaussian envelope. Its reaction with sodium dithionite leads to a quenching of the radical and a loss of the EPR signal.

The setup had to be slightly modified to reach faster time points by reducing the delay line volume from 35 µL employed for the PSII experiments (Figure S3) to 3.5 µL. The desired delay time was then obtained via precise control of the flow rate (see Table S2). These estimations show the laminar flow of the mixture through the delay line and the nozzle (Re < 2300) at all given time points. The pressure buildup at the 10 ms time point is already reaching 30 bar, and a further increasing flow rate would rapidly inflate the pressure that may be damaging in case of biologic samples.

**Table S2**. Calculated flow rates, linear velocity, back pressure, filling time and corresponding Reynolds numbers for glycerol/water 10% v/v solution.

| | | *Q (ml/s)* | *v (m/s)* | *ΔP (bar)* | *t (ms)* | *Re* |
|---|---|---|---|---|---|---|
| *Delay line* | | 0.35 | 6.9 | 26.9 | 10 | 1303 |
| *i.d.* | 0.254 mm | 0.18 | 3.5 | 6.7 | 20 | 652 |
| *len.* | 70 mm | 0.12 | 2.3 | 3.0 | 30 | 434 |
| *S* | 0.05 mm$^2$ | 0.09 | 1.7 | 1.7 | 40 | 326 |
| *Vol.* | 3.5 µL | 0.07 | 1.4 | 1.1 | 50 | 261 |
| *Nozzle* | | 0.35 | 15.8 | 30.4 | 0.6 | 1970 |
| *i.d.* | 0.168 mm | 0.18 | 7.9 | 7.6 | 1.3 | 985 |
| *len.* | 10 mm | 0.12 | 5.3 | 3.4 | 1.9 | 657 |
| *S* | 0.02 mm$^2$ | 0.09 | 3.9 | 1.9 | 2.5 | 493 |
| *Vol.* | 0.2 µl | 0.07 | 3.2 | 1.2 | 3.2 | 394 |

The sample packing procedure through the collection funnel is far from optimal, particularly in case of narrow inner diameter tubes/capillaries (W-band EPR tubes). This is a known issue with freeze quench measurements and requires the addition of a standard. The $Mn^{2+}$ salt ($MnCl_2$) was used in this experiment, which being dissolved in solution corresponds to $Mn(H_2O)_6$. This was added to the TEMPO reactant and allowed the TEMPO concentration to be robustly estimated. The EPR spectrum of $Mn(H_2O)_6$ represents a six-line signal shown in red. All data were scaled to the background $Mn(H_2O)_6$ signal.

The reaction follows pseudo-first order kinetics with an effective reaction constant in good agreement with the literature value (2), demonstrating the mixing is effective and that the performance of the mixing device is well calibrated.

**S1.3 *Calibration and performance testing of µRFQ apparatus – long timescale regime (>100 ms to seconds).*** To test the performance of the apparatus over the 100 ms to seconds range the reductant was switched to sodium ascorbate in excess (**Fig. S6**). As before, a $Mn^{2+}$ salt ($MnCl_2$) was added as an internal standard.

The specifications for all HPLC tubing in this configuration for W-band samples was as follows: all lines used O.D. 1/16 inch ETFE HPLC tubing (I.D. = 0.508 mm and 0.762 mm), with the mixing and exit line having a 15 µL and 35 µL volume, respectively.

For convenience, the measurements were performed in both W-band and Q-band EPR tubes (for details, see **Table S1**). For Q-band, two sets were prepared: the first having an initial sample amount of 60 µL (labelled high vol Q-band; black dots in **Fig. S6**) and a second set having an initial volume of 30 µL (labelled low vol Q-band; red stars in **Fig. S6**), i.e. exactly the same conditions as for W-band (blue stars in **Fig. S6**). Importantly, the same TEMPOL signal, scaled to the reference, was observed regardless of tube size or initial sample volume.

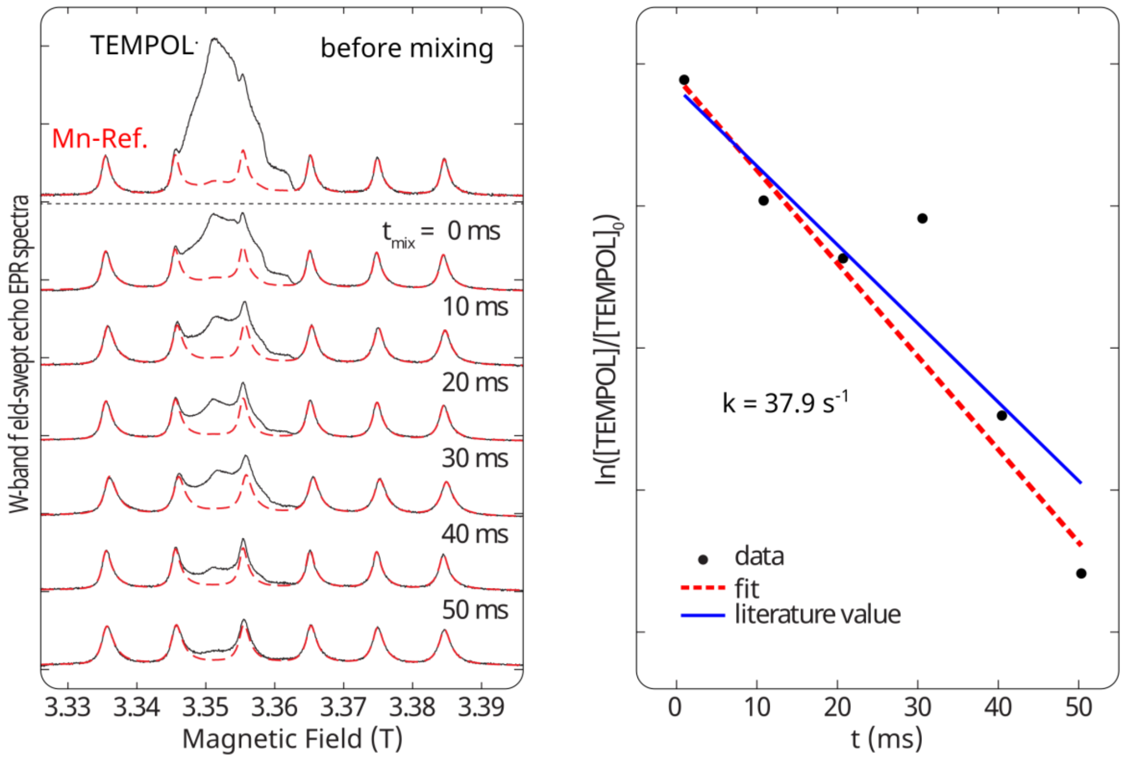


**Fig. S5**: Calibration of short mixing times via the reaction of TEMPOL with sodium dithionite as monitored using pulse W-band EPR. **Left:** EPR spectra of TEMPOL (black) and the $Mn^{2+}$ standard (dashed-red) before (top) and after various mixing times with sodium dithionite. $MnCl_2$ was added to the TEMPOL solution as reference for normalization. **Right:** Change of TEMPOL concentration with mixing times. Concentrations before mixing: [TEMPOL] = 2 mM; [$MnCl_2$] = 0.4 mM; and [NaDT] = 100 mM, both in 140mM phosphate buffer, pH 7.0, 10% v/v glycerol/water.

As before, the reaction followed pseudo-first order kinetics (**Fig. S6**) with an effective reaction constant 1.65 $s^{-1}$, demonstrating the mixing is effective and that the performance of the mixing device is well calibrated. Additionally, the interception of the fitted line with the abscissa gives an estimation for the freezing time, which is around 110 ms.

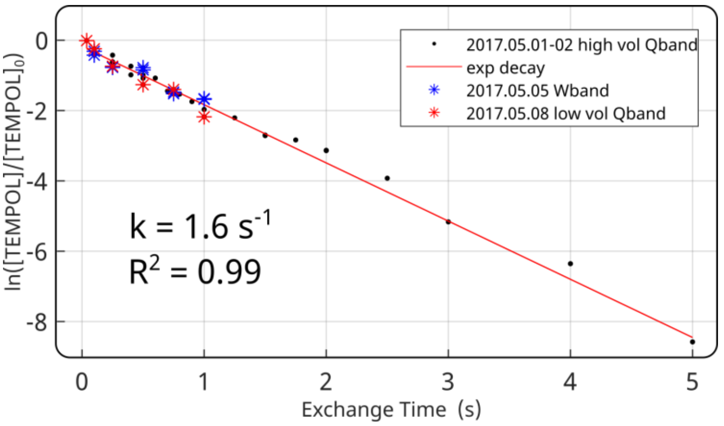


**Fig. S6**: The reaction of TEMPOL with sodium ascorbate as monitored using pulse EPR. $MnCl_2$ was added to the TEMPOL solution as reference for normalization. Comparison of kinetics for different data sets: black dots – high volume Q-band samples; blue stars – W-band samples; red stars – low volume Q-band samples. Concentrations before mixing: [TEMPOL] = 2 mM; [$MnCl_2$] = 0.4 mM; and [NaAsc] = 0.5 M (at pH 10.0).

**S1.4** ***Practical mixing times achieved for protein samples.*** The minimum practical mixing time achieved for the target protein, PSII, was 100 ms. This was set by the volume of the exit line and the flow rate. These two factors are additive, because the flow rate was limited to reduce the tubing backpressure. A low backpressure was required, because at higher pressures the protein sample decomposed. This could be assessed by monitoring the background tyrosine D radical ($Y_D^{\bullet}$).

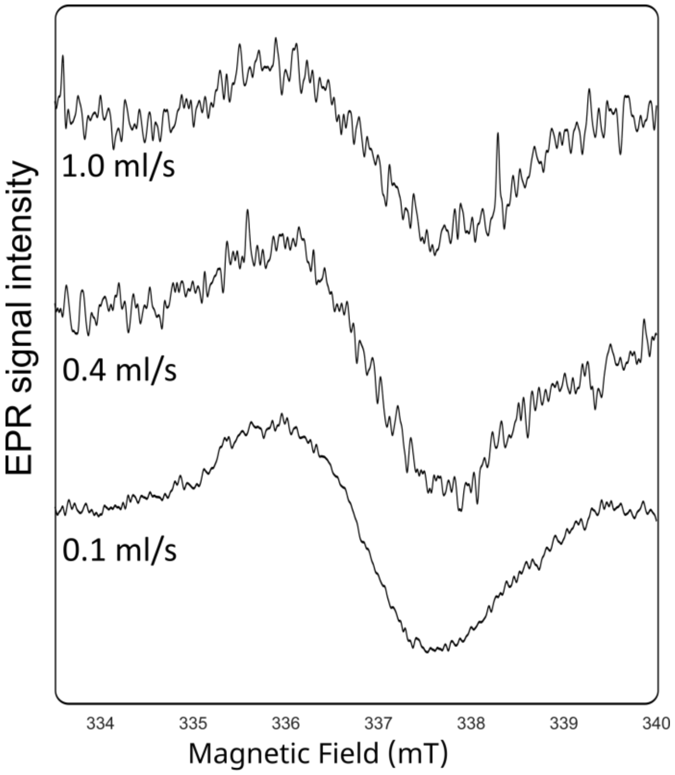


**Fig S7**. The X-band CW EPR spectra of the $Y_D^{\bullet}$ radical measured at 173K. The EPR spectra are plotted with an offset and normalized to the m.w. power. Note the signal is overmodulated, resulting in the loss of hyperfine structure.

A $Y_D^{\bullet}$ radical is observed in all functional PSII protein supercomplexes which receive a pre-illumination. For these experiments, pre-illumination consisted of loading the sample into the µRFQ instrument under dim green light. As $Y_D^{\bullet}$ is not part of the functional electron transport chain, its intensity does not change during the S-state cycling measurements described, here allowing it to be used as an internal standard.

At the flow rates up to 0.4 ml/s, in the presence of 10% glycerol, the concentration of $Y_D^{\bullet}$ did not noticeably change as compared to control samples (here the signal is divided by 2 to adjust for dilution). If, however, the flow rate was increased to 1.0 ml/s, around 20% of the $Y_D$ signal was lost (**Fig. S7**). The pressure experienced by the protein was estimated in Table S3 using the equation for the straight pipe. Here the glycerol/water 10% v/v solution was taken as the model system. In fact, the protein samples are even more viscous meaning the pressure buildup is underestimated.

**Table S3**. Calculated linear velocity, back pressure, filling time and Reynolds numbers for glycerol/water 10% v/v solution.

| | | *Q (ml/s)* | *v (m/s)* | *ΔP (bar)* | *t (ms)* | *Re* |
|---|---|---|---|---|---|---|
| *Delay line* | | 1 | 2.2 | 1 | 35 | 1241 |
| *i.d.* | 0.762 mm | 0.4 | 0.9 | 0.16 | 88 | 496 |
| *len.* | 77 mm | 0.1 | 0.2 | 0.01 | 351 | 124 |
| *Vol.* | 35 µL | | | | | |
| *Nozzle* | | 1 | 45 | 248.1 | 0.2 | 5629 |
| *i.d.* | 0.168 mm | 0.4 | 18 | 39.7 | 0.6 | 2252 |
| *len.* | 10 mm | 0.1 | 4.5 | 2.5 | 2.2 | 563 |
| *Vol.* | 0.2 µL | | | | | |

PSII deactivation, however, may not be a direct consequence of the pressure but may instead reflect increased sample heating due to friction. Based on this estimation, the final flow rate of 0.4 ml/s was selected for the further experiments on PSII as seemingly best compromise between speed of mixing and intactness of the PSII sample.

## S2. Materials and Methods

**2.1 *PSII core complex preparations***. PSII core complexes were prepared from *Thermosynechococcus (T.) vestitus* (previously known as *T. elongatus*) as described previously (3-7). Two preparations were used: a wild type (WT*) and a His-tag strain. Control EPR data measured on PSII core complexes isolated from either strain were identical. The His-tag strain was used for samples in which $Ca^{2+}$ was biosynthetically exchanged with $Sr^{2+}$ (3), since Sr-PSII is only stable in the presence of betaine, which is incompatible with the intermediate HIC step of the $Ca^{2+}$ standard isolation protocol. This step was thus replaced by an IMAC His-tagged purification method for the purification of His-tagged PSII containing $Sr^{2+}$ (3). The final buffer of Ca preparations contains: 500 mM mannitol, 40 mM MES (pH = 6.5), 10 mM $CaCl_2$, 10 mM $MgCl_2$, 0.03% v/v n-dodecyl β-D-maltoside and for Sr: 1.2 M betaine, 20 mM MES (pH = 6.5), 10 mM $CaCl_2$, 10 mM $MgCl_2$, 0.03% v/v n-dodecyl β-D-maltoside, 10% v/v glycerol. PSII core complexes were concentrated to a Chl concentration of 2-3 mg Chl $ml^{-1}$ and stored at -80° C until use.

**2.2 *Freeze quench sample preparation for W-band EDNMR.*** PSII samples were thawed and concentrated to either 4-5 mg Chl mL-1 (EDNMR set 1) or 8-9 mg Chl $mL^{-1}$ (EDNMR set 2) using Millipore microcentrifuge filters. Under dim green light, the modified BioLogic QFM-400 mixing module was prepared for freeze quench mixing (see **Fig S2** for schematic of mixing module). Volume V1 was loaded with the PSII sample, V2 was loaded with either 77% $H_2{}^{17}O$ enriched buffer (EDNMR set 1) or with pure 86% $H_2{}^{17}O$ (EDNMR set 2), while V3 was loaded with buffer. 15 µL PSII and 15µL $^{17}O$-labelled buffer (or water) were mixed and loaded into a 30 µL delay line, where it was incubated for a given time, before being pushed out through a nozzle to generate a mist. A surrounding stream of $N_2$ gas was employed to guide the aerosolized PSII into an aluminum collection unit cooled with liquid $N_2$. The collection unit contained a feeding funnel leading directly to the W-band EDNMR tube, situated below in the liquid $N_2$, **Fig 2B**. The resulting frozen sample was crushed on the cold surface with a spatula to a fine powder and packed into the W-band EDNMR tube (i.d. 0.6 mm, o.d. 0.84 mm) using an optic fiber (o.d. 0.5 mm). A typical W-band EDNMR sample contained around 4 µl of dark-green frozen particles. Timing and volumes for the rapid freeze quench sequence were controlled from the BioKine software and triggered through an MPS 70 control unit.

To advance the PSII sample into the $S_2$ state for EDNMR measurements, samples were illuminated with continuous light at 200 K for 3 seconds.

**2.3 *W-band pulse EPR measurements*** were performed using a Bruker ELEXSYS E680 spectrometer equipped with a homebuilt W-band bridge and a cryogen free Cryogenic 6 T magnet (J4322) with a variable temperature insert. All measurements shown in this work were recorded at $T = 4.8$ K. Electron spin echo (ESE)-detected field-swept spectra were measured using the pulse sequence: $t_p$–$\tau$–$2t_p$–$\tau$–echo. The length of the $\pi/2$ microwave pulse was generally set to $t_p$ = 20 ns and the interpulse distance was set to $\tau$ = 600 ns. Electron spin nutation curves were measured using the pulse sequence: $t_{prep}$–T–$t_p$–$\tau$–$2t_p$–$\tau$–echo. The preparation pulse length ($t_{prep}$) was incremented over the range of 2-1000 ns in 2 ns steps. The length of the $\pi/2$ microwave pulse was $t_p$ = 20 ns and the interpulse delays were T = 1.0 µs and $\tau$ = 600 ns. ELDOR-detected NMR (EDNMR) spectra were collected using the pulse sequence: $t_{HTA}$–T–$t_p$–$\tau$–$2t_p$–$\tau$–echo (8). The high turning angle (HTA) microwave pulse was applied at microwave frequency $\nu_{mw}$. The detection pulse sequence $t_p$–$\tau$–$2t_p$–$\tau$–echo, applied at the microwave frequency matching the cavity resonance, was set at T = 6 µs after the HTA pulse to ensure near-complete decay of the electron spin coherencies. The $\pi/2$ pulse length used for detection was $t_p$ = 80 ns, and the inter-pulse separation was $\tau$ = 600 ns. The echo was integrated over 600 ns centered at its maximum. The spectra were acquired by continuously sweeping the HTA frequency $\nu_{mw}$ at a fixed magnetic field in steps of 0.097 MHz. The length and amplitude of the HTA microwave pulse were set to 5 µs and $\omega_1 = 4.7 \times 10^7$ rad.$s^{-1}$ ($\Delta\nu_{1/2} \approx 15$ MHz), respectively.

**2.4 *TR-MIMS experiments*** were performed in a stirred, temperature controlled (20ºC) membrane-inlet cell (165 µl volume), connected to a magnetic sector field isotope ratio mass spectrometer (ThermoFinnigan Delta$^{Plus}$ XP) via a cooling trap (liquid $N_2$), as previously described (9, 10). Prior to loading, the samples were diluted to 0.3 mg Chl $ml^{-1}$ and pre-flashed with a single saturating flash from a Xenon-flash lamp (5 µs FWHM) and dark-adapted for 1 hour at room-temperature. After dark adaptation, 2,5-dichloro-1,4-benzoquinone was added from a 200 mM stock in dimethyl-sulfoxide (DMSO) to a final concentration of 0.2 mM. The sample was then loaded under dim green light into the membrane-inlet cell and degassed for 20 minutes while stirring. For measuring the water exchange in the $S_1$ state, the samples were rapidly enriched with $H_2{}^{18}O$ (97%, 8 ms mixing time) and three additional flashes were applied to generate $O_2$ with spacings of 10 ms and 20 ms between the two first and two last flashes, respectively. The delay between the isotopic enrichment and the flash sequence was incremented from 8 ms to 60 s. The final $^{18}O$

sample enrichment was 22%. Molecular oxygen dissolved in the $H_2{}^{18}O$ was removed inside the delivery syringe (modified Hamilton CR-700-50) by glucose/glucose-oxidase and catalase (9). After 5 minutes, 4 additional flashes were given at 2 Hz and the oxygen yield induced was used for normalization. Data analysis was performed as previously described (9, 10), see also SI text S3.2.

**Table S4.** Buffer composition during water exchange in the $S_1$ state. All additions taken into account, including labelled water.

| *Experiment* | *pH* | *MES (mM)* | *mannitol (mM)* | *betaine (mM)* | *glycerol (%, v/v)* | *$CaCl_2$ (mM)* | *$MgCl_2$ (mM)* | *Deter.[1] (%, w/v)* | *DMSO[2] (%, v/v)* |
|---|---|---|---|---|---|---|---|---|---|
| *Ca-PSII* | | | | | | | | | |
| ***MIMS[3]*** | 6.5 | 18 | 375 | - | - | 7.5 | 7.5 | 0.020 | 0.075 |
| ***EDNMR[4]*** | 6.5 | 20 | 275 | - | 10 | 10 | 10 | 0.030 | - |
| ***EDNMR[5]*** | 6.5 | 10 | 250 | - | - | 5 | 5 | 0.015 | - |
| *Sr-PSII* | | | | | | | | | |
| ***MIMS[3]*** | 6.5 | 15 | - | 900 | 0.75 | 7.5 | 7.5 | 0.02 | 0.075 |
| ***EDNMR[4]*** | 6.5 | 20 | 25 | 600 | 10 | 10 | 10 | 0.030 | - |

*[1]n-dodecyl β- maltoside; [2]plus 0.2 mM DCBQ (2,5 dichloro-benzoquinone); [3]sample concentration: 0.3 mg Chl/ml; [4]set1 (2.0-2.5 mg/ml Chl); [5]set2 (4.0-4.5 mg/ml Chl)*

## S3 Data Analysis

***3.1 Analysis of EDNMR data***. As discussed briefly in the main text, here we only performed a simple decomposition of the $^{17}O$ EDNMR signals as compared to the original publication (11, 12). A detailed motivation for this is given here: In **Figure 1F**, two species are readily observed: i) a background doublet centered at the $^{14}N$ Larmor frequency ($\nu(^{14}N)$ = 10.5 MHz at 3.4 T) assigned to the only nitrogen ligand of the cofactor, His332, and a broader envelope centered at the $^{17}O$ Larmor frequency assigned to exchanged oxygen sites of the cofactor (12). The profile is dependent on the initial pulse length/amplitude, indicating the envelope is made up of multiple different $^{17}O$ species. In our original study (12), the profile was fitted as three distinct signals: i) a broad signal, which describes the width of the envelope. This was assigned to a single, exchanged oxygen bridge (O5), based on comparisons to model systems, and by chemical modification of the cofactor (11-14); ii) a narrow matrix signal, assigned to exchanged water ligands of the cofactor, dominantly W1; and iii) a third signal with intermediate width that is hidden under the signal envelope but is better resolved in the double quantum region. This was assigned to a terminal hydroxide ligand of Mn4, W2 (12). Treatment of PSII with ammonia confirms this assignment. $NH_3$ displaces W1 leading to the suppression of the $^{17}O$ matrix signal, with the central peak broadening as it now has a larger contribution from W2 (11).

It is important to note that the exact contributions of O5 and W2 to the profile cannot be experimentally determined. In our original study, the three species were each modelled as two Gaussians of different peak separation (hyperfine splitting) (12). This, however, is only an approximation. As we demonstrated in subsequent publications studying model systems (14), the oxygen bridge signal is likely to significantly contribute to the center of the spectrum, owing to its hyperfine interaction tensor having a large anisotropic contribution (with unresolved quadrupole splitting), and as such, is better modelled as a rectangular envelope centered at the $^{17}O$ Larmor frequency, as opposed to two discrete peaks (14). The same is likely true for the W2 signal i.e., it too will resemble a broad, albeit narrower, unstructured envelope, based on comparison to the terminal water ligand of Mn catalase, which displays approximately the same hyperfine coupling (14, 15), see **Figure S8.** Note that the water ligand of Mn catalase has a hyperfine coupling that is very similar to OH ligand (W2) in PSII. This is because the spin density of the catalase cofactor is asymmetrically distributed – the spin projection factor for the $Mn^{III}$ in the catalase is 2.

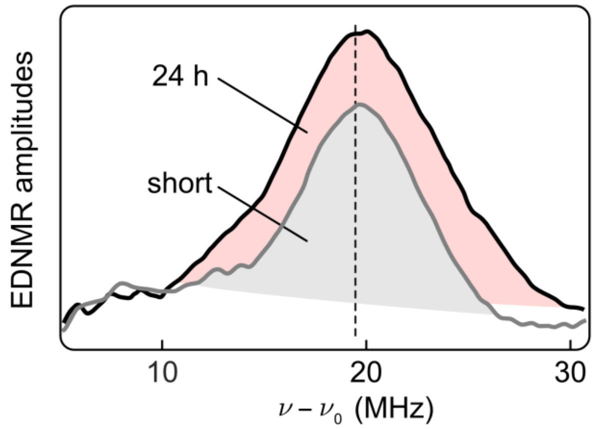


**Fig. S8.** $^{17}O$ EDNMR signal envelope seen for Mn Catalase poised in the III/IV oxidation state. The Mn catalase has two exchangeable oxygen ligands: i) a terminal water ligand, with hyperfine coupling similar to that inferred for W2; and ii) an exchangeable μ-oxo bridge. Unlike O5 in PSII, the exchangeable μ-oxo bridge exchanges on a much slower timescale of the order of hours. Redrawn after Fig 6B in ref. (14).

As described in the main text, the intensity of EDNMR signals is dependent on the experimental setting (HTA pulse) used. This explains why the $^{17}O$ EDNMR profile reported in this study is slightly different from that of our earlier work. As compared to these earlier studies (12), the $^{14}N$ and the central $^{17}O$ peak are somewhat suppressed relative to the O5 bridge signal. This is because of the choice of the initial HTA pulse that was chosen in this study to best observe the O5 signal. We note that this should also lead to the W2 having a larger contribution to the center of the $^{17}O$ signal envelope, at the expense of W1. A larger W2 contribution to the central peak may explain why this peak is structured in some of our datasets (see **Fig. S9**).

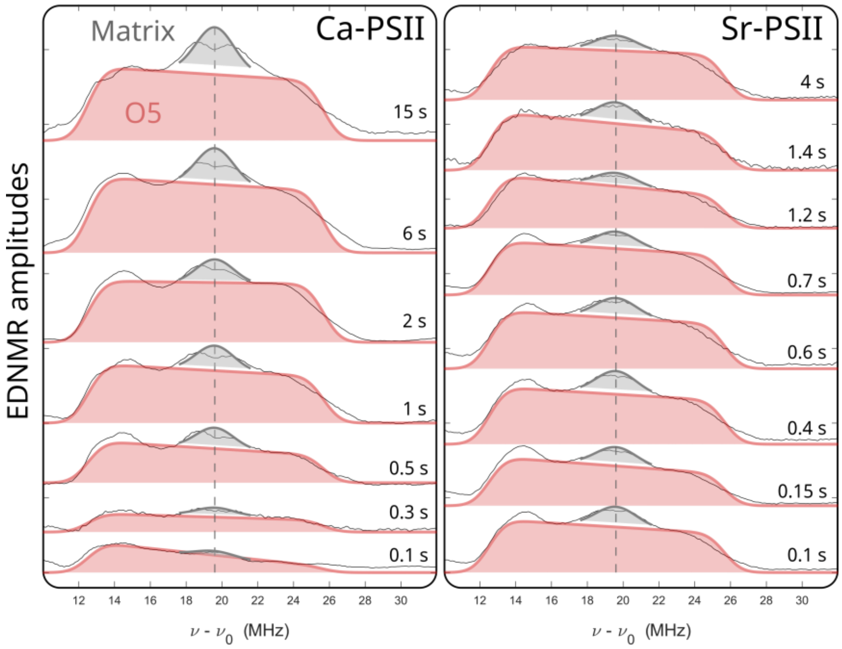


**Fig. S9.** An independent $^{17}O$ TR-EDNMR Ca-PSII dataset (Ca-Set2) next to the full Sr-PSII dataset (Sr-Set1), partially presented in the main text.

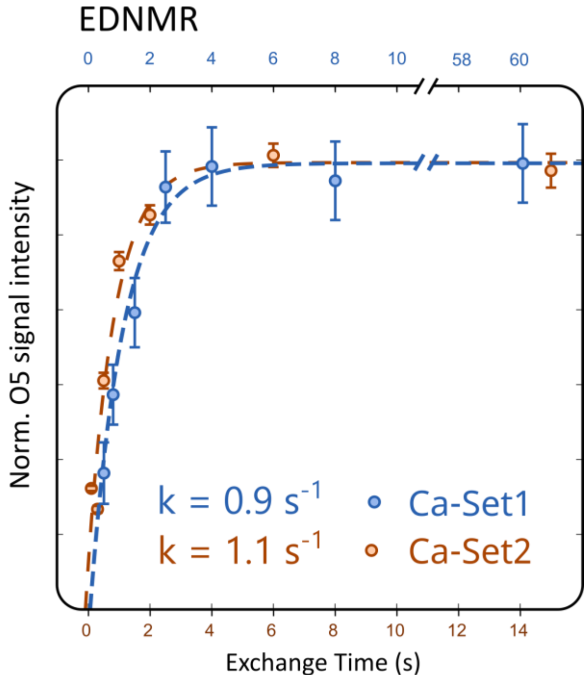


**Fig. S10.** Kinetics curves for the rise of the O5 signal in two independently obtained TR-$^{17}O$-EDNMR datasets: blue – the Ca-Set1 presented in the main text, dark orange – the Ca-Set2 (Fig. S9). Note that the time axis for data set 1 is given in blue on top (broken time axis), while that for data set 1 is given in brown at the bottom. Error bars represent the integrated fitting error.

The Ca-PSII experiment was repeated (Fig. S9, left), resulting in a very similar kinetic rate for O5 exchange (see comparison in **Fig. S10**).

***3.2 Analysis of TR-MIMS.*** The substrate exchange rate $k_s$ of the slow substrate water was determined by a simultaneous fit of the *m*/*z* 34 (**Figure S11**) and the *m*/*z* 36 data (**Figure 3C**) to equations (1) and (2), as described in (10). Since the fast exchange was not resolved, the fit could be reduced to only include the amplitudes of their exchange phases (***a*** and ***b***). The parameter ***a*** is the ratio of the fast and slow exchange phases in the $^{34}O_2$ data. The parameter ***a*** was determined from the initial enrichment ($\alpha_{in}$) and the final enrichment ($\alpha_f$) as shown in equation (3). The initial enrichment was found to be slightly elevated (0.07%) over natural enrichment as a result of leakage from the syringe tip. For the Sr-PSII exchange, an extra exponential phase was needed to fit the data as described in (10). The amplitude of this phase was given by the factor *b*, which was determined by a fit to the m/z 36 data and held constant in the global fit. The factor ***b*** was set to 0 for Ca-PSII.

$$m/z\,34 = a + (1-a)\cdot\left(b \cdot +(1-b)\cdot\left(1-e^{-k_s \cdot t}\right)\right) \quad (1)$$

$$m/z\,36 = b \cdot +(1-b)\cdot\left(1-e^{-k_s \cdot t}\right) \quad (2)$$

$$a = \frac{\alpha_f \cdot (1-\alpha_{in}) + (1-\alpha_f)\cdot \alpha_{in}}{(1-\alpha_f)\cdot \alpha_f \cdot 2} \quad (3)$$

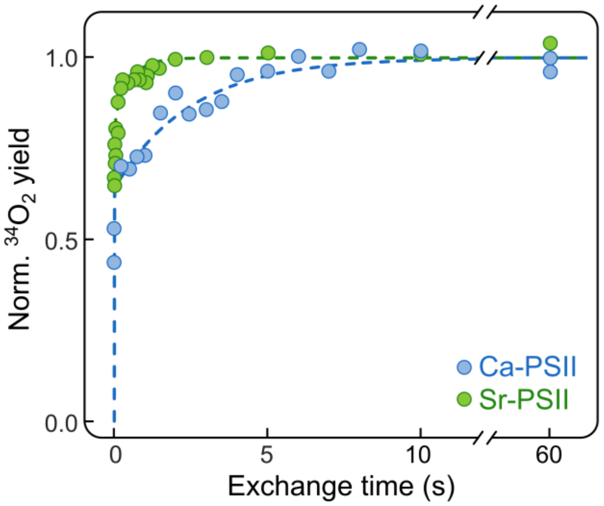


**Figure S11:** Normalized $^{34}O_2$ flash yields of Ca-PSII (blue) and Sr-PSII (green) core complexes of *T. vestitus* induced by three saturating flashes after different exchange times with $H_2{}^{18}O$ at 20°C and pH 6.5. Each dot represents a separate measurement. The dashed lines display fits of the exchange rates of the fast and slow substrate waters ($W_f$ and $W_S$). Fits to Equations 1- 3 imply an unresolved $W_f$ exchange and resulted in a $W_S$ exchange rate of 0.4 $s^{-1}$ (Ca-PSII) and of 1.9 $s^{-1}$ (Sr-PSII).

***3.3 Estimation of temperature dependence of water exchange in the $S_1$ state.***

The temperature dependence of the water exchange can be estimated employing the Eyring equation

$$k = (k_B T/h) \times e(-\Delta G^{\ddagger} /RT)$$

where $k_B$ is the Boltzmann constant, T the absolute temperature, h the Planck constant, R the gas constant and $\Delta G^{\ddagger}$ the Gibbs energy of activation.

The rate of $W_S$ was determined at 20°C (293K) to be 0.4 $s^{-1}$, which corresponds to a barrier of about $\Delta G^{\ddagger}$ = 17.7 kcal/mol (or 74 kJ/mol). This estimate is likely a lower bound, since previously a similar barrier has been determined experimentally for the water exchange in the $S_3$ state of spinach thylakoids that is about 10-fold faster than the exchange in the $S_1$ state (9, 16). At such activation energy, a moderate temperature increase to 28-30°C is enough to explain the difference in rate that we observed. Given that the room temperature was 22°C during the EDNMR experiments, transient warming of the samples during the rapid mixing process by as little as 6-8°C is needed to explain the small elevation in exchange rate compared to the MIMS experiments performed at 20°C.

**References**


1. V. Schünemann *et al.*, Tyrosine radical formation in the reaction of wild type and mutant cytochrome P450cam with peroxy acids: A multifrequency EPR study of intermediates on the milisecond time scale. *J. Biol. Chem.* **279**, 10919-10930 (2004).
2. A. Potapov, D. Goldfarb, A calibration reaction for rapid freeze-quench W-band EPR. *Appl. Mag. Res.* **37**, 845-850 (2010).
3. A. Boussac *et al.*, Biosynthetic $Ca^{2+}/Sr^{2+}$ exchange in the photosystem II oxygen-evolving enzyme of *Thermosynechococcus elongatus*. *J. Biol. Chem.* **279**, 22809-22819 (2004).
4. H. Kuhl *et al.*, Towards structural determination of the water-splitting enzyme: purification, crystallization and preliminary crystallographic studies of photosystem II from a thermophilic cyanobacterium. *J. Biol. Chem.* **275**, 20652-20659 (2000).
5. M. M. Nowaczyk *et al.*, Psb27, a cyanobacterial lipoprotein, is involved in the repair cycle of photosystem II. *The Plant Cell* **18**, 3121-3131 (2006).
6. N. Ishida *et al.*, Biosynthetic exchange of bromide for chloride and strontium for calcium in the photosystem II oxygen-evolving enzymes. *J Biol Chem* **283**, 13330-13340 (2008).
7. M. Chrysina *et al.*, Five-coordinate Mn-IV intermediate in the activation of nature's water splitting cofactor. *Proc. Natl. Acad. Sci. USA* **116**, 16841-16846 (2019).
8. P. Schosseler, T. Wacker, A. Schweiger, Pulsed ELDOR detected NMR. *Chem. Phys. Lett.* **224**, 319-324 (1994).
9. J. Messinger, M. Badger, T. Wydrzynski, Detection of *one* slowly exchanging substrate water molecule in the $S_3$ state of photosystem II. *Proc. Natl. Acad. Sci. USA* **92**, 3209-3213 (1995).
10. C. de Lichtenberg, J. Messinger, Substrate water exchange in the $S_2$ state of photosystem II is dependent on the conformation of the $Mn_4Ca$ cluster. *Phys. Chem. Chem. Phys.* **22**, 12894-12908 (2020).
11. M. P. Navarro *et al.*, Ammonia binding to the oxygen-evolving complex of photosystem II identifies the solvent-exchangeable oxygen bridge (μ-oxo) of the manganese tetramer *Proc. Natl. Acad. Sci. USA* **110**, 15561-15566 (2013).
12. L. Rapatskiy *et al.*, Detection of the water-binding sites of the oxygen-evolving complex of photosystem II using W-band $^{17}O$ electron-electron double resonance-detected NMR spectroscopy. *J. Am. Chem. Soc.* **134**, 16619-16634 (2012).
13. T. Lohmiller *et al.*, Structure, ligands and substrate coordination of the oxygen-evolving complex of photosystem II in the $S_2$ state: a combined EPR and DFT study. *Phys. Chem. Chem. Phys.* **16**, 11877-11892 (2014).
14. L. Rapatskiy *et al.*, Characterization of oxygen bridged manganese model complexes using multifrequency $^{17}O$-hyperfine EPR spectroscopies and density functional theory. *J. Phys. Chem. B* **119**, 13904-13921 (2015).
15. I. L. McConnell *et al.*, EPR-ENDOR characterization of ($^{17}O$, $^{1}H$, $^{2}H$) water in manganese catalase and its relevance to the oxygen-evolving complex of photosystem II. *J. Am. Chem. Soc.* **134**, 1504-1512 (2012).
16. W. Hillier, J. Messinger, T. Wydrzynski, Kinetic determination of the fast exchanging substrate water molecule in the $S_3$ state of photosystem II. *Biochemistry* **37**, 16908-16914 (1998).
